# 

**Published:** 1921-01

**Authors:** 


### SUBJECT INDEX TO VOLUME LXXV

EVENT AND COMMENT

After the Vacation. By C. N. Johnson, 534.

An Appeal to the Dental Profession, 505.

Anatomy as Applied to Oral Surgery and Block Anesthesia. By Arthur E. Smith, 523.

Are Dental Diseases Curable? 201.

Classified Index for Dental Literature, 101.

Commercialism, 579.

Dangers from Oral Infections, 201.

Honor to Doctor Charles N. Johnson, 252.

National Dental Association, 504.

New School Prosthodontia, 451.

Preserving the Pulps, 1.

Proposes Plan for the Dental Education of the Public, 401.

Prosthetic Technique, 51.

Standardizing or Drifting? 151.

Technical Skill and Its Limitations, 551.

The Danger of Extremes in Dental Practice, 251.

The Dental Economic Problem, 501.

The National Dental Association, 504.

What Further is Demanded of Dentistry in View of Our Present Standards and Practices? 35.

FORMAL PAPERS

A Plan for the Ethical Dental Education of the Public. By W. Linford Smith, 402.

A Review of Certain Experimental Findings Upon the Accessory Food Factors. By John A. Marshall, 269.

A Review of Pyorrhea Alveolaris. By M. Rattner, D.D.S., 304.

A Step in Advance. By Julio Endelman, 130.

Banquet to C. N. Johnson, 507.

Cast Construction for Artificial Dentures. By I. Lester Furnas, 282.

Chays Movable-Removable Bridgework. By Walter G. Hine, 174.

Closer Affiliation Between Medicine and Dentistry. By E. C. Kirk, 426.

Co-operation in Preparing Mouths for Dentures. By Frank Mihnos, D.M.D., 265.

Crown and Bridge Work. By R. E. MacBoyle, 517.

Danger Signals in Nitrous Oxide Oxygen Anesthesia. By E. Roy Bier, 510.

Dental Office Efficiency and the Application. By Harry J. Bosworth, 21.

Dentifrices. By C. Edmund Kells, 452.

Discriminating Prophylaxis. By Julio Endelman, 267.

Granulation and Differential Diagnosis of Favorable and Unfavorable Cases. By Thomas P. Hinman, 153.

Impacted Lower Third Molar. By C. Edmund Kells, 218.

Improving the Practice of Prosthetic Dentistry. By M. F. Lasby.

Industrial Dentistry. By Carey P. McCord, 30.

Industrial Dentistry. By J. P. Becker, 76.

Instruction of Patients in the Personal Care of the Mouth. By Justin D. Towner, 322.

Local Anesthesia. By J. H. Anderson, L.D.S., 179.

Local Anesthesia in Every-day Conservative Practice. By Alexander Naismith, L.D.S., 186.

Modeling Compound Impression Taking. By Edward Kennedy, 472.

Modern Dentistry for the Laity. By A. A. Crocker, 100.

Mouth Hygiene. By Guy S. Millberry, 415.

Need and Value of Demonstrations to Assure Success in Amalgam Work. By Wm. E. Harper, 554.

Oral Infection. By A. W. Thornton, 278.

Origin of Modern Dentistry for the Laity, 200.

Partial Dentures. By I. J. Dresch, 480.

Perfecting the Plaster Cast. By I. L. Furnas, 576.

Periodontia. By P. R. Thomas, D.D.S., 11.

Porcelain Removable Bridge with Gum Pink Porcelain Saddle. By Joseph A. Carey, 274.

Possibilities of the Callahan Method. By H. B. Johnson, 202.

Pre-Dental Year in Dental Education, 437.

Preventive Orthodontia. By C. M. McCauley, 527.

Problems in Diagnosis. By Julio Endelman, 573.

Public Dental Dispensaries. By Julio Endelman, 159.

Relation of Food to Developing Teeth. By M. Evangeline Jordon, 563.

Retention of Full Upper and Lower Dentures. By Russell W. Tench, 211.

"Salesmanship in Dentistry": A Critical Inquiry. By Edward C. Kirk, 236.

Salesmanship in Dentistry. By H. G. Frankel, 345.

Secondary Dentine and Cementum. By W. Clyde Davis and R. Ottolengui, 53.

Should All Pulpless and Impacted Teeth be Removed. By W. H. G. Logan, 163.

Some of the Present Tendencies in Dentistry. By C. N. Johnson, 254.

Standardization of Nitrous Oxid-Oxygen, Anesthesia Induction. By J. A. Heidbrink, 367.

Suppose You Retired from Business Now? By O. F. Lewis, 134.

The Cause and Treatment of Chronic Infections Involving the Teeth. By F. B. Moorehead, 356.

The Des Moines Toothbrush Lecture. By C. M. Kennedy, 431.

The Diagnosis of Peridontal Diseases. By Russel W. Bunting, 406.

The Future of Bridge Restorations. By James K. Burgess, 61.

The Future of Dentistry. By Eugene H. Smith, 549.

The New Dental Graduate. By Eugene H. Talbot, 353.

The Patient's Point of View. By C. N. Johnson, 581.

The Present Day Tendencies of Dental Practice. By L. P. Anthony, D.D.S., 16.

The Relation of the General Practitioner to the Specialist in Dentistry. By A. W. Thornton, D.D.S., 3.

The Relations of Dental Sepsis to Other Pathologic Lesions. By Frank Colyer, F.R.C.S., 369.

Unveiling Monument to Dr. F. H. Rehwinkel, 103.

Value of Co-operation of Dentist and Banker. By C. W. Banta, 117.

Western Reserve Takes Important Step in Dental Education, 439.

What Shall We Tell the People? By R. P. McGee, 422.

What's in a Name? 332.

Why Members of the Dental Profession Should Not be Classed as Manufacturers. By R. G. McLaughlin, 57.

INDENTS

Abnormal Calcification, 39.

Acid vs. Alkaline Dentifrices, 583.

Bachelor of Dental Surgery, 299.

Berlin Dental Clinic Closed, 499.

Better Express Service, 146.

Bicarbonate of Soda for Sensitive Teeth, 37.

Bonwill Mechanical Mallet, 250.

Cancer Phobia, 543.

Cast Upper Denture with Reference to Anatomy and Function of the Soft Tissues, 462.

Casting a Band or Crown, 48.

Caution for Radicular Operations, 41.

Cavity Preparation, 450.

Cementing an Inlay, 244.

Chewing Gum, 544.

Children in Practice, 455.

Classification of Dental Schools, 96.

Cleaning Aluminum Plates, 289.

Cleaning Gold Work After Polishing, 289.

Clinical Aspect of the Dental Situation, 138.

Conservatism in Extracting, 494.

Conservative Dentistry, 338.

Contact Points of Silicate Fillings, 244.

Correcting Artificial Teeth, 340.

Cotton Rolls, 490.

Dangers in the Use of Adrenalin, 288.

Dead Teeth and Devitalized Teeth, 140.

Death from Aplastic Anemia, 297.

Defective Teeth Among School Children, 137.

Dental Caries as Affecting Gout and Rheumatism, 491.

Dental Ideals, 298.

Dentist, Know Your Banker, 487.

Development of Health Specialists, 42.

Do Not Use Lubricants on Silicous Fillings, 244.

Don't Extract Without Reason, 585.

Don't Use Ammonia to Remove Silver Nitrate Stains, 198.

Effectiveness of Tooth Washes, 294.

Etching the Surface of a Gold Inlay, 246.

Ethanesal, 296.

Ethical Parables, 293.

Ethical Parables, 343.

Ethyl-Chloride Spray for Inflammation and Pain, 466.

Eulogy of Henry W. Morgan, 41.

Extraction and Eradication of Diseased Periapical Tissues, 399.

Filling Small Cavities, 38.

Fishers' Maxims, 37.

Gum Chewing a Bad Habit, 538.

Gum Tissue Grafting, 245.

Harmless Gingivitis, 395.

Immediate Extractions and Denture Insertions, 584.

Importance of Oral Hygiene During Childhood, 539.

Important Decision, 589.

Indiscriminate Tooth Extraction, 87.

Instruction in Dental Hygiene. By H. W. Guthrie.

Intellectual Securities, 298.

Internal Revenue Income Tax, 92.

Interstitial Gingivitis, An Incipient Form of Scurvy. By E. S. Talbot, 492.

Ivy, R. H., 400.

Lecture on the Teeth. By A. A. Crocker, 147.

Let There Be Light, 290.

Lighting Gas Without a Match, 296.

Make Your Own Flux, 199.

Maxillary X-Ray Landmarks, 448.

More Frequent Consulations, 537.

Mouth Wash, 393.

Narrow Bicuspids in Bridge Work, 246.

Nevada Uses Gas for Legal Death, 394.

New Regulations for Narcotic Drugs, 541.

Novocain a Skin Irritant, 91.

Novocain a Skin Irritant, 341.

Only a Dentist, 400.

Oral Hygiene and Preventive Dentistry, 588.

Oral Hygiene for Students, 300.

Oral Hygiene School Physiologies, 337.

Oral Prophylaxis, 90.

Packing Vulcanite, 587.

Partial Denture vs. Removable Bridge, 393.

Periodontoclasia, 540.

Physical Exercise and the Middle-Aged Man, 247.

Pine Products as Disinfectants, 540.

Pink Rubber Inset, 589.

Porcelain Pontics, 144.

Post-Operative Pain, 194.

Prevent Checking Porcelain in Casting, 296.

Prophylactic Aid, 294

Quick Repair of Porous Plates, 344.

Refining Wax, 350.

Refit an Upper Vulcanite Plate, 145.

Refitting Dentures, 583.

Restoring Lower Incisors, 244.

Rochester Dental Dispensary, 395.

Roughen a Broach, 145.

Rustless Steel, 38.

Scrutinizing Collection Agencies Contracts, 447.

Sensitive Dentine, 38.

Shall Pulpless Teeth be Retained in the Mouth? 531.

Sore Mouths from Vulcanite Plates, 336.

Spare the Tooth and Soil the Fad, 240.

Stop Leakage in Hypodermics, 246.

Study of Pulps from Infected Teeth, 490.

Sulphuric Acid and Potassium in Root-Canal Cleaning, 294.

Surgeon's Soap, 587.

Synthetic Fillings, 295.

Taking a Bite for Crown or Bridge Work, 447.

Taking the Bite for Full Upper and Lower Dentures, 493.

The Circle of Industrial Service, 350.

The Dental Educational Council of America, 86.

The Dual Nature of Apothesine, 299.

The Office Coat, 297.

The Question of Amalgam, 143.

Tobacco Smoke as a Disinfectant, 248.

To Clean Modeling Compound Trays, 393.

To Freshen Gold for Casting, 288.

To Grasp Broaches, 589.

Toothbrush a Cause of Mouth Infection, 393.

Tooth Decay Under Clasps, 397.

Trigeminal Neuralgia, 29.

Two Dentists I know, 489.

Use of Dental Floss, 290.

Who is Joseph Lobosco? 350.

Wire Clasp vs. Cast Clasp, 88.

### BIBLIOGRAPHY

Block Anesthesia. By. Dr. A. E. Smith, —.

Electro-Radiographic Diagnosis. By Howard R. Raper, 592.

Lincoln's Life and Sketches. By Dr. Newkirk, 98.

### OBITUARY

Mathew H. Cryer, 545.

Robert A. Molynaux, 249.

### ANNOUNCEMENTS

American College of Dentists, 495.

American Institute of Dental Teachers, 146, 591.

Dental Protective Association, 546.

Dewey Orthodontia School Alumni Association, 50, 99.

Kentucky State Dental Society, 55, 99, 591.

Massachusetts State Dental Society, 99.

Michigan State Board Meeting, 495.

Minnesota State Dental Society, 546.

National Dental Association, 25.

Ohio State Dental Society, 546.

Tennessee State Dental Association, 99.

Texas, Annual State Convention, 55.

Texas Dental Society, 590.

U. S. Civil Service Examination, 546.

Western Pennsylvania Odontological Society, 99.

BIOGRAPHIC

Adachi, S., 290.

Allshouse, C. [illegible]., 450.

Anthony, L. P., 16.

Banta, C. W., 129.

Barkley, G. C., 589.

Becker, J. P., 76.

Bier, E. Roy, 510.

Blum, T., 340.

Bogle, R. Boyd, 41.

Bosworth, H. J., 21.

Bremner, M. D., 545.

Briggs, J. S., 296.

Broomell, J. N., 298.

Bruce, Wm. I., 297.

Bunting, R. W., 406, 467.

Burch, R. W., 490.

Burgess, J. K., 60.

Butler, H. B., 540.

Carey, J. A., 274.

Clapp, Geo Wood, 294, 343, 489.

Cleveland, Carlton, 480.

Cobb, C. M., 393.

Colyer, F., F.R.C.S., 369.

Crocker, A. A., 100, 147, 200.

Cruis, R. J., 244.

Davis, W. Clyde, 53.

DeLane, F. E., 246.

Dittmar, G. W., 467.

Dr. Conzette, 47.

Dr. Door, 494.

Dr. Kennedy, 337.

Dr. Levy, 492.

Dresch, I., 480.

Ellman, F. O., 247.

Endelman, Julio, 130, 159, 269, 397, 573.

Evans, E. T., 145.

Evans, Dr. W. A., 44.

Faison, C. I., 344.

Felcher, F. R., 340.

Fine, Wm. M., 494.

Fones, A. C., 587.

Frahm, F. H., 294.

Frankel, H. G., 345.

Furnas, I. L., 282, 576.

Guptill, A. E., 92, 341.

Guthrie, H. W., 47.

Hadley, C. J., 246.

Harper, W. E., 554.

Heidbrink, J. A., 387.

Henrici and Hartzell, 490.

Hine, W. G., 174.

Hinman, T. P., 88, 153.

Hoyt, W. H., 588.

Hyatt, T. B., 589.

Johnson, C. N., 1, 254, 300, 534, 582.

Johnston, H. B., 202.

Jordon, M. E., 563.

Jordon, W. H., 90.

Kauffman, J. H., 91.

Kells, C. E., 218, 452.

Kennedy, C. M., 431.

Kennedy, E., 472.

Kirk, E. C., 236, 322, 426.

Lasby, M. F., 313.

LeMyre, Charles, 289, 290.

Levin, G., 296.

Lewis, O. F., 134.

Lindsay, G. R., 540.

Logan, W. H. G., 163.

MacBoyle, R. E., 517.

MacMillan, J. J., 246.

McCauley, C. M., 527.

McCord, C. P., 30.

McGee, R. P., 422.

McLaughlin, R. G., 57.

Marguilies, I., 393.

Marshall, John A., 269.

Millberry, Guy S., 415.

Molyneaux, R. A., 249.

Moore, W. D., 244, 288.

Moorehead, F. B., 356.

Mowbra, F. W., 262.

Muir, R. A., 289.

Naismith, A., 186.

Nelson, A. A., 394.

Nelson, G. F., 296.

Oral Hygiene, 337.

Ottolengui, R., 53.

Park Davis Notes, 300.

Peters, M. E., 536.

Raper, H. R., 592.

Rattner, M., 304.

Rehwinkel, F. H., 103.

Rogers, A. P., 446.

Rose, J. E., 145.

Rosenthal, J. M., 587.

Search, J. T., 145.

Seccomb, Wallace, 539.

Schlieffarth, H. F., 447.

Smedley, V. C., 584.

Smith, A. E., 244, 523.

Smith, Eugene H., 549.

Smith, W. L., 402.

Stansbery, C. J., 244.

Stephan, F. W., 446.

Talbot, Eugene H., 353, 492.

Tench, R. W., 211.

Thomas, P. R., 11.

Thornton, A. W., 3, 269.

Thwaits, W. H., 450.

Towner, J. D., 322.

Turner, C. K., 298.

Underdahl, L., 398.

Viverito, J. A., 393.

Williams, T. C., 589.

### ANNOUNCEMENTS

---

Annual Convention Texas State Dental Socciety. The Texas State Dental Society will hold its forty-first annual convention on Thursday, Friday and Saturday, the 3rd, 4th and 5th of March, 1921, at Dallas, Tex.

The Executive Committee is endeavoring to make this meeting an unusually attractive one, and to that end will not only utilize the best talent of the society, but will bring in a few outside men of note.

Clinics will be made a prominent feature.

On Monday following will commence the society's second annual post-graduate class course, to continue one week, and which will be conducted by the following instructors: Dr. Rupert E. Hall, Chicago; Dr. F. Ewing Roach, Chicago; Dr. T. W. Maves, Minneapolis; Dr. Arthur E. Smith, Chicago; Dr. Thos. B. Hartzell, Minneapolis; Dr. Clarence O. Simpson, St. Louis.—J. G. Fife, Secy., Dallas, Tex.

---

Kentucky State Dental Association. The next annual meeting of the Kentucky State Dental Association will be held in Louisville, Ky., April 13, 14 and 16, 1921, the Seelbach Hotel as headquarters. A clinical program of unusual interest is being arranged. Address all correspondence to W. M. Randall, Secy.

---

Alumni Society of the Dewey School of Orthodontia. The next annual meeting of this society will be held on April 25th and 26th at the Hotel Ambassador in Atlantic City. The usual high standard of the meetings of this society will be maintained. Clinics and evening sessions will be included in the program. All interested in orthodontia are cordially invited to attend these meetings. —Geo. F. Burke, Secy., 741-43 David Whitney Bldg., Detroit, Mich.

